# Reversed urban–rural gradient in COVID-19 seroprevalence and related factors in a nationally representative survey, Poland, 29 March to 14 May 2021

**DOI:** 10.2807/1560-7917.ES.2023.28.35.2200745

**Published:** 2023-08-31

**Authors:** Michał Czerwiński, Małgorzata Stępień, Grzegorz Juszczyk, Małgorzata Sadkowska-Todys, Adam Zieliński, Jakub Rutkowski, Magdalena Rosińska

**Affiliations:** 1 National Institute of Public Health NIH–National Research Institute (NIPH NIH–NRI), Warsaw, Poland; 2 Department of Public Health, Medical University of Warsaw, Warsaw, Poland

**Keywords:** COVID-19, SARS-CoV-2, seroprevalence, rural areas, urban-rural gradient, OBSER-CO, Poland

## Abstract

**Background:**

We anticipated that people in rural areas and small towns with lower population density, lower connectivity and jobs less dependent on social interaction will be less exposed to COVID-19. Still, other variables correlated with socioeconomic inequalities may have a greater impact on transmission.

**Aim:**

We investigated how COVID-19 affected rural and urban communities in Poland, focussing on the most exposed groups and disparities in SARS-CoV-2 transmission.

**Methods:**

A random digit dial sample of Polish adults stratified by region and age was drawn from 29 March to 14 May 2021. Serum samples were tested for anti-S1 and anti-N IgG antibodies, and positive results in both assays were considered indicative of past infection. Seroprevalence estimates were weighted to account for non-response. Adjusted odds ratios (AORs) were calculated using multivariable logistic regression.

**Results:**

There was serological evidence of infection in 32.2% (95% CI: 30.2–34.4) of adults in rural areas/small towns (< 50,000 population) and 26.6% (95% CI: 24.9–28.3) in larger cities. Regional SARS-CoV-2 seroprevalence ranged from 23.4% (95% CI: 18.3–29.5) to 41.0% (95% CI: 33.5–49.0) and was moderately positively correlated (R = 0.588; p = 0.017; n = 16) with the proportion of respondents living in rural areas or small cities. Upon multivariable adjustment, both men (AOR = 1.60; 95% CI: 1.09–2.35) and women (AOR = 2.26; 95% CI: 1.58–3.21) from these areas were more likely to be seropositive than residents of larger cities.

**Conclusions:**

We found an inverse urban–rural gradient of SARS-CoV-2 infections during early stages of the COVID-19 pandemic in Poland and suggest that vulnerabilities of populations living in rural areas need to be addressed.

**Key public health message**

**What did you want to address in this study?**

We wanted to know whether the risk of COVID-19 was different between rural and urban areas during the initial phases of the pandemic. We focused on identifying socio-demographic groups where SARS-CoV-2 infection spreads more efficiently.

**What have we learnt from this study?**

Residents of rural communities and small cities in Poland were at an increased risk of SARS-CoV-2 infection. Both men and women from these areas were more likely to have antibodies against SARS-CoV-2 than residents of larger cities.

**What are the implications of your findings for public health?**

It is likely that new virus variants will also continue to spread faster through rural communities in Poland. Therefore, these populations may require specific surveillance measures and particularly adjusted risk communication strategies.

## Introduction

The COVID-19 pandemic in 2020 and 2021 affected different populations unequally due to pre-existing disparities in socioeconomic status, demographics and health-related factors, as well as living and environmental conditions [1,2]. These differences are likely to exist even within countries or regions, and they may be associated with a socioeconomic gradient [3]. Living in crowded conditions, not being able to work remotely or implement physical distancing measures in the workplace, and having less access to healthcare or trust in public health communications could all contribute to higher exposure to severe acute respiratory syndrome coronavirus 2 (SARS-CoV-2) associated with lower socioeconomic status [4].

According to mathematical modelling, airborne outbreaks are thought to spread faster and reach higher levels in cities due to higher population density and denser contact networks [5]. In fact, models predict a high correlation between the size of an outbreak and population connectivity in a given area [5]. However, the socioeconomic status in some large cities, especially metropolitan areas in Europe, is higher than in rural areas, and the latter may therefore be more vulnerable to the COVID-19 epidemic [6]. Furthermore, observational studies do not always confirm the simple urban–rural gradient. Reports from Bangladesh, France, Iran and the United States (US) showed that the urban population had a higher number of cases, while studies from China, Italy and the Netherlands found an insignificant effect of urbanisation on COVID-19 spread [2]. In addition, some reports showed that in some regions of the US in 2020, the intensity of the epidemic shifted from urban to rural areas, possibly due to disparities in socioeconomic determinants of health [4,7].

The first case of COVID-19 in Poland was confirmed on 4 March 2020, and a state of epidemic emergency was declared. Mitigation measures included mobility restrictions, physical distancing and mask wearing, suspending mass and indoor gatherings, closing non-essential businesses and schools and encouraging remote work. These generalised restrictions were gradually lifted starting from 20 April 2020 [8], to be replaced in August by specific regional restrictions based on 14-day infection rates. Nonetheless, in autumn and winter, in response to a surge in new cases, further nationwide restrictions were reintroduced and while they were amended over time, most of the rules remained in force until May 2021.

The highest reported 14-day incidence of COVID-19 during the 2020 epidemic waves in Poland varied between 627 and 1,162 per 100,000 [9] with no apparent pattern, although the peak was in the highly urbanised region of Silesia in November. Already released research has consistently found that the true underlying incidence in Poland was 2–10 times higher than reported rates [10-12]. The high testing positivity rate, which typically exceeded 20% during the wave in autumn 2020, suggests that cases were vastly undercounted [13].

Taking into account possible biases in case ascertainment, we aimed to estimate the risk of infection during the early stages (approximately the first year) of the COVID-19 pandemic based on a representative nationwide seroepidemiological survey of adults in Poland. The study was conducted between 29 March and 14 May 2021, during the third wave and 4 months after the autumn (second) wave. We focused on rural–urban differences in the spread of SARS-CoV-2 infections and identified groups and characteristics that were associated with an increased risk of SARS-CoV-2 infection and that contribute to these disparities.

## Methods

### Study design

This study was part of the OBSER-CO survey, designed as a cross-sectional study using a nationwide sample of Polish adults (≥ 20 years-old) from each administrative region (voivodeship) and age category, in line with the World Health Organization (WHO)-Unity protocol: *Population-based age-stratified seroepidemiological investigation protocol for COVID-19 infection* [14]. The survey model entailed telephone recruitment and an interview, followed by laboratory testing with a participation code distributed during the interview. It was conducted during the first stage of the COVID-19 immunisation programme. By the study's first week, 14.3% of the adults in Poland eligible for vaccination, i.e. healthcare workers, seniors over 60, nursing home residents, teachers and members of the uniformed services, had been immunised with at least one dose of a COVID-19 vaccine. By the study's final week, when immunisation was available to all adults, this percentage had increased to 32.7%.

### Sampling

The survey sample was drawn using landline and mobile random digit dial (RDD) sampling and stratified by voivodeship and age group (20–39, 40–59, 60–69 and ≥ 70 years). Three attempts were made to reach each of the sampled numbers. Survey participation rates were tracked, collecting the reason for non-participation, if feasible.

### Interview

Interviews were performed with consenting respondents who met the socio-demographic inclusion criteria (age and place of residence), which were modified throughout the study to generate a proportionate sample for each stratum. Data were collected using computer-assisted telephone interviewing (CATI) by trained interviewers. The survey questions were developed in the Department of Epidemiology and Surveillance of the National Institute of Public Health based on the questionnaire appended to the WHO Unity Protocol [14]. The questionnaire was not validated but consisted of items from existing tools or items already used in a previous seroprevalence study [12].

### Laboratory methods

One of Poland's largest networks of medical laboratories, with 627 specimen collection facilities located in all areas of the country, collected venous blood samples and performed serological testing for anti-SARS-CoV-2 antibodies. During the telephone interview, each participant received a unique code valid for 2 weeks, and reminders were sent via cell phone text message to participants who did not present for testing during the first week after the interview. The specimens were collected between 29 March and 14 May 2021.

IgG antibodies against the spike protein S1 domain were measured using a quantitative enzyme immunoassay (Euroimmun Anti-SARS-CoV-2 QuantiVac ELISA). The manufacturer's reference range was used to interpret the results: negative result (−): < 25.6 binding antibody units (BAU)/mL; borderline result (+/−): 25.6–35.2 BAU/mL; positive result (+): > 35.2 BAU/mL.

To distinguish natural from vaccine-induced immunity, sera from vaccinated participants were also examined for the presence of anti-nucleocapsid IgG as a marker of natural infection using semiquantitative ELISA (Euroimmun Anti-SARS-CoV-2 NCP–IgG). A specimen was classified as negative (−) if the ratio was less than 0.8; borderline (+/−) if the ratio was between 0.8 and 1.01; and positive (+) if the ratio was greater than 1.01. Threshold values were adopted according to the test manufacturer's reference range.

### Statistical analysis

The intended sample size was calculated based on binomial proportion for each of the region–age group stratum (16 regions and four age groups) in order to achieve a coefficient of variation of 30% with seroprevalence of 35%, a confidence level of 5% and power of 80%. This yielded a sample size of 79 per stratum and a total sample size of 5,056. We assumed that 20% of individuals would attend tests after RDD recruitment, so the sample size for the RDD recruitment step should be 25,280.

The SARS-CoV-2 seroprevalence was calculated as the proportion of respondents who had evidence of past SARS-CoV-2 infection. Evidence of past infection was defined as the detection of (i) anti-S1 IgG SARS-CoV-2 antibodies (positive anti-SARS-CoV-2 QuantiVac ELISA) among unvaccinated individuals or (ii) anti-nucleocapsid IgG SARS-CoV-2 IgG (positive anti-SARS-CoV-2 NCP-IgG) among people seropositive for anti-S1 IgG, i.e. previously vaccinated individuals. Information on vaccination was obtained from the survey, based on the respondent's declaration. Records with borderline laboratory results were excluded, but we performed a sensitivity analysis to determine robustness of the observed reversed urban–rural gradient. The detailed replication of the results with all borderline records retained in the analysis and recoded against the gradient is appended in the Supplement.

We employed a two-stage adjustment to our SARS-CoV-2 seroprevalence estimates to account for non-response at the level of the telephone interview and at the level of presentation for testing via inverse probability weighting. The final weight was applied to records of respondents with valid test results, and it was calculated as a product of stage 1 and stage 2 weights. Applying individual weights to each CATI respondent who submitted a specimen allowed us to calculate the SARS-CoV-2 seroprevalence estimates unaffected by selection bias due to observed confounders. Details on the design of this adjustment, the development of statistical weights and replication of the major findings without weights are provided in the Supplement.

Confidence intervals (CI) for binomial proportion were calculated using the logit transform method for the weighted estimates of the proportions, as implemented in the svy: prop STATA 14 command (StataCorp LLC). Differences in weighted proportions between characteristics of respondents living in less and more urbanised areas were assessed using the default (in STATA) test of independence for survey data, i.e. the chi-squared statistic corrected with the second-order correction for the survey design and converted into an F statistic.

Multivariable analysis sought to examine effect modification, i.e. a two-way interaction with a dichotomised place of residence and risk factors for COVID-19 infection with subject matter importance. To force a complete assessment of all possible two-way interactions with the place of residence in a multivariable logistic model, we used a backward elimination method. The Supplement provides a description of the development process of the multivariable model.

The final multivariable logistic model retained two statistically significant interactions (involving place of residence and sex coded as a binary variable; place of residence and household size) and the following nine main effects: region, age, sex, place of residence, household size, have worked during restrictions, have worked mainly remotely, contact with a known COVID-19 case, and have received at least one dose of a vaccine against COVID-19.

## Results

### Socio-demographic characteristics

A flowchart with a summary of the enrolment process is provided in Supplementary Figure S1.

Of the 271,827 randomly dialled phone numbers, 223,875 met the inclusion criteria, of whom 25,812 (11.5%) agreed to be interviewed. Our study population comprised a subset of 25,202 CATI respondents aged 20 years or older, of whom 5,892 provided a blood sample for anti-SARS-CoV-2 antibody testing. Upon weighting, serosurvey participants were comparable to CATI respondents and the general population distribution. Supplementary Tables S1 and S2 list the sociodemographic characteristics of the study subjects compared with the target population.

Of the 5,892 respondents included in the analysis, 2,803 lived in rural areas or small towns with fewer than 50,000 people and 3,089 in mid-sized or major cities with populations of 50,000 or more. Overall, people in rural or small-town areas were more likely to live in households with five or more members (19.3% vs 7.2%; p < 0.001) and to cohabit with at least one child under the age of 18 years (39.9% vs 29.5%; p < 0.001). This tendency was especially noticeable among respondents aged 20–39 years. People in rural or small-town areas were also less likely to be working mainly remotely since March 2020 (12.3% vs 21.0%; p < 0.001) and more likely to be unemployed (9.7% vs 6.4%; p < 0.001). See Supplementary Table S3 for the detailed model data.

### Seroprevalence of SARS-CoV-2 infections in the total sample

Of the 5,892 people tested for IgG SARS-CoV-2 antibodies, 3,207 (54.0%) tested positive for anti-S1 IgG and 1,859 (31.6%) had previous SARS-CoV-2 infection. The overall weighted seroprevalence of the past infection estimate was lower, at 29.8% (95% CI: 28.4–31.2).

**Figure 1:**
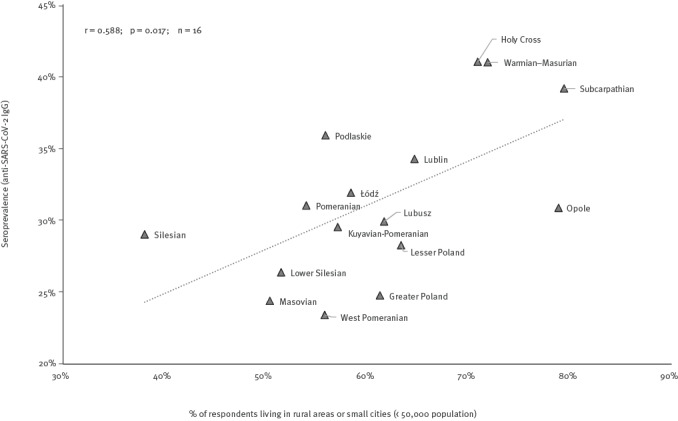
Correlation between proportion of respondents from rural areas/small cities^a^ and seroprevalence estimates of anti-SARS-CoV-2 IgG antibodies (past infections) in the voivodeship, OBSER-CO cross-sectional survey, Poland, 29 March–14 May 2021 (n = 5,892)

^a^ Rural areas/small cities were those with a population < 50,000.

The seroprevalence of SARS-CoV-2 infections varied substantially by region of the country, ranging from 23.4% (95% CI: 18.3–29.5) in the West Pomeranian to 41.0% (95% CI: 33.5–49.0) in Holy Cross Voivodeship, and was moderately positively correlated (R = 0.588; p = 0.017; n = 16) with the proportion of respondents living in rural areas or small cities (Figure 1).

**Figure 2:**
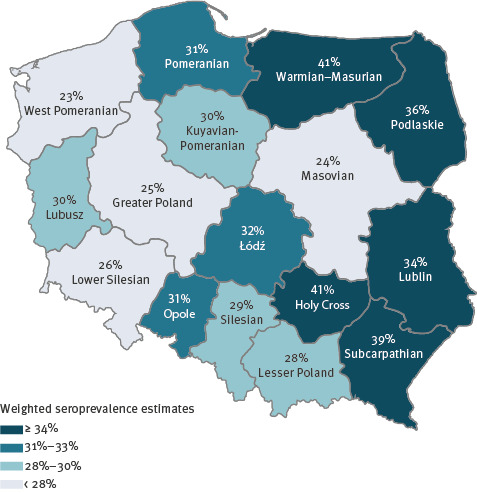
Weighted seroprevalence estimates of anti-SARS-CoV-2 IgG antibodies (past infections) by voivodeship, OBSER-CO cross-sectional survey, Poland, 29 March–14 May 2021 (n = 5,892)

Overall, Eastern Poland – a macroregion comprised of Lublin, Subcarpathian, Podlaskie, Holy Cross and Warmian–Masurian Voivodeships – was the most affected part of the country, with an estimated prevalence of 38.0% (95% CI: 34.8–41.4) (Figure 2).

### Prevalence of SARS-CoV-2 infections by socio-demographic characteristics and associated factors

Overall, respondents living in rural areas or small cities tended to have a higher prevalence of SARS-CoV-2 infection than residents of mid-sized or large cities (32.2%; 95% CI: 30.2–34.4 vs 26.6%; 95% CI: 24.9–28.3) (Table 1).

**Table 1:** Estimates of anti-SARS-CoV-2 IgG seroprevalence (past infections) by socio-demographic characteristics, OBSER-CO cross-sectional survey, Poland, 29 March–14 May 2021 (n = 5,892)

Population/ characteristic	n tested	Past SARS-CoV-2 infections			
		n positive	Seroprevalence estimates^a^	Univariate OR (95% CI)	Multivariate AOR (95% CI)^b^
Total sample					
Age group (years)					
20–39	1,392	412	29.1%	Reference	
40–59	2,163	757	32.9%	1.19 (1.00–1.41)	1.34 (1.12–1.60)
60–69	1,424	438	26.4%	0.87 (0.73–1.05)	1.80 (1.43–2.27)
≥ 70	913	252	28.0%	0.95 (0.75–1.19)	2.82 (2.06–3.86)
Sex					
Female	3,455	1,098	30.6%	1.08 (0.94–1.24)	See subgroups
Male	2,437	761	28.9%	Reference	
Place of residence					
Rural	1,406	504	33.2%	1.41 (1.20–1.65)	Not applicable
Urban < 50,000	1,397	463	30.2%	1.23 (1.05–1.44)	
Urban 50,000–100,000	490	154	29.4%	1.18 (0.94–1.50)	
Urban > 100,000	2,599	738	26.0%	Reference	
Place of residence combined					
< 50,000 population	2,803	967	32.2%	1.31 (1.15–1.50)	See subgroups
≥ 50,000 population	3,089	892	26.6%	Reference	
Subgroups					
Areas < 50,000 population					
Female	1,637	595	34.4%	1.24 (1.02–1.51)	1.34 (1.10–1.65)
Male	1,166	372	29.7%	Reference	
Areas ≥ 50,000 population					
Female	1,818	503	25.4%	0.88 (0.74–1.05)	0.95 (0.79–1.15)
Male	1,271	389	27.9%	Reference	
Female					
< 50,000 population	1,637	595	34.4%	1.54 (1.30–1.82)	2.26 (1.58–3.21)
≥ 50,000 population	1,818	503	25.4%	Reference	
Male					
< 50,000 population	1,166	372	29.7%	1.09 (0.89–1.33)	1.60 (1.09–2.35)
≥ 50,000 population	1,271	389	27.9%	Reference	

CI: confidence interval; AOR: adjusted odds ratio; OR: odds ratio; SARS-CoV-2: severe acute respiratory syndrome coronavirus 2.

^a^ Weighted seroprevalence estimates calculated using the final weights, $w_{k}$ (see the definition in the Supplement).

^b^ The OR was adjusted in the final multivariable logistic model. Because of the significant interaction between sex and place of residence, the effect of sex is presented separately for rural areas/small cities (< 50,000) and mid-sized/large cities (≥ 50,000), just as the effect of place of residence is presented separately for males and females.

**Figure 3:**
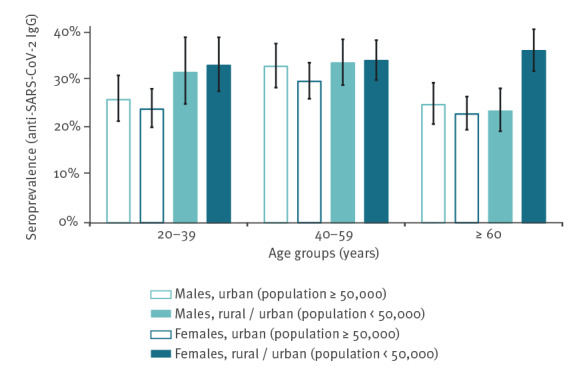
Weighted seroprevalence estimates of anti-SARS-CoV-2 IgG antibodies (past infections) stratified by sex, age and place of residence, OBSER-CO cross-sectional survey. Poland, 29 March–14 May 2021 (n = 5,892)

Upon age and sex stratification, this difference was noteworthy for the age group 20–39 years (Figure 3). Stratified seroprevalence was clearly more equally distributed among respondents aged 40–59 years. Among older respondents aged 60 and older, women living in rural areas or small cities had the highest stratified IgG SARS-CoV-2 seroprevalence (36.2%; 95% CI: 31.9–40.7) in the sample.

In multivariable analysis, living in rural areas or small cities was significantly associated with SARS-CoV-2 seropositivity even after adjusting for region of the country, age, sex, COVID-19 vaccination status and other factors associated with SARS-CoV-2 infection (Table 1). The final multiple logistic model also indicated a strong interaction between sex and place of residence. The model revealed that women living in rural areas or small cities had overall higher odds of SARS-CoV-2 infection compared with males in the same areas (AOR = 1.34; 95% CI: 1.10–1.65), but in cities with 50,000 or more people, there were no sex differences in SARS-CoV-2 seropositivity (AOR = 0.95; 95% CI: 0.79–1.15). On the other hand, both women and men living in rural areas or small cities had overall higher odds of SARS-CoV-2 infection compared with those from mid-sized or large cities, with AOR respectively 2.26 (95% CI: 1.58–3.21) and 1.60 (95% CI: 1.09–2.35).

Upon adjustment for COVID-19 vaccination status, the overall odds of SARS-CoV-2 seropositvity also increased with age. In addition, the multivariable logistic model revealed statistically significant interaction between place of residence and household size (p = 0.002) (Table 2).

**Table 2:** Household related risk factors for SARS-CoV-2 IgG antibodies (past infections) in OBSER-CO cross-sectional survey, Poland, 29 March–14 May 2021 (n = 5,892)

Population	n tested	n positive	Seroprevalence estimates^a^	Univariate OR (95% CI)	Multivariate AOR (95% CI)^b^
Total sample					
Cohabiting with a child younger than 18 years					
No	4,030	1,203	28.5%	Reference	Not applicable
Yes	1,862	656	32.1%	1.18 (1.03–1.36)	
Household size					
1 additional resident in a household				1.15 (1.09–1.22)	See subgroups
1 person in a household	950	242	25.1%	0.75 (0.59–0.94)	
2 persons in a household	2,171	625	26.2%	0.79 (0.66–0.95)	
3 persons in a household	1,087	372	31.0%	Reference	
4 persons in a household	1,093	385	33.5%	1.12 (0.90–1.38)	
≥ 5 persons in a household	591	235	35.9%	1.25 (0.97–1.60)	
Subgroups					
Areas < 50,000 population					
1 additional resident in a household				1.06 (0.99–1.15)	1.04 (0.95–1.13)
1 person in a household	332	103	31.2%	0.95 (0.67–1.36)	0.92 (0.64–1.33)
2 persons in a household	947	309	29.3%	0.87 (0.67–1.13)	0.95 (0.72–1.26)
3 persons in a household	560	201	32.3%	Reference	
4 persons in a household	569	201	34.2%	1.09 (0.82–1.46)	1.09 (0.81–1.48)
≥ 5 persons in a household	395	153	34.4%	1.10 (0.80–1.52)	1.04 (0.74–1.45)
Areas ≥ 50,000 population					
1 additional resident in a household				1.26 (1.17–1.36)	1.24 (1.14–1.35)
1 person in a household	618	139	21.2%	0.66 (0.49–0.88)	0.67 (0.49–0.91)
2 persons in a household	1,224	316	23.1%	0.74 (0.57–0.94)	0.79 (0.61–1.03)
3 persons in a household	527	171	29.0%	Reference	
4 persons in a household	524	184	32.1%	1.16 (0.87–1.53)	1.09 (0.82–1.46)
≥ 5 persons in a household	196	82	41.3%	1.72 (1.18–2.51)	1.89 (1.26–2.82)
1-person households					
< 50,000 population	332	103	31.2%	1.69 (1.18–2.43)	1.34 (1.00–1.80)
≥ 50,000 population	618	139	21.2%	Reference	
2-person households					
< 50,000 population	947	309	29.3%	1.38 (1.10–1.72)	1.12 (0.89–1.41)
≥ 50,000 population	1,224	316	23.1%	Reference	
3-person households					
< 50,000 population	560	201	32.3%	1.16 (0.87–1.55)	0.94 (0.76–1.16)
≥ 50,000 population	527	171	29.0%	Reference	
4-person households					
< 50,000 population	569	201	34.2%	1.10 (0.83–1.46)	0.78 (0.61–1.00)
≥ 50,000 population	524	184	32.1%	Reference	
*≥* 5-persons households					
< 50,000 population	395	153	34.4%	0.75 (0.5–1.12)	0.65 (0.47–0.90)
≥ 50,000 population	196	82	41.3%	Reference	

CI: confidence interval; AOR: adjusted odds ratio; OR: odds ratio; SARS-CoV-2: severe acute respiratory syndrome coronavirus 2.

^a^ Weighted seroprevalence estimates calculated using the final weights, $w_{k}$ (see the definition in the Supplement)

^b^ The OR was adjusted in the final multivariable logistic model. Because of the significant interaction between household size and place of residence, the effect of household size is presented separately for rural areas/small cities (< 50,000) and mid-sized/large cities (≥ 50,000), just as the effect of place of residence is presented separately for each category of household size.

While household size was a significant predictor for IgG SARS-CoV-2 seropositivity in mid-sized and large cities, it had no effect in rural areas or small cities. Similarly, the odds of seropositivity increased significantly (AOR = 1.24; 95% CI: 1.14–1.35) with each additional resident in a household. As a consequence, in mid-sized or large cities, the highest stratified seroprevalence was seen in residents of households with five or more members (41.3%; 95% CI: 33.8–49.2). In contrast, the lowest seroprevalence was found among respondents who were living alone or cohabiting with only one person (22.4%; 95% CI: 20.3–24.6). These trends were not seen in rural areas or small cities, where seropositivity was more evenly distributed across household sizes.

Overall, respondents who worked throughout the pandemic had significantly higher odds of seropositivity (Table 3).

**Table 3:** Work-related and other risk factors for SARS-CoV-2 IgG antibodies (past infections) in OBSER-CO cross-sectional survey, Poland, 29 March–14 May 2021 (n = 5,892)

Factor	n tested	n (+)	Seroprevalence estimates^a^	Univariate OR (95% CI)	Multivariate AOR (95% CI)^b^
Work-related exposures					
Employed/self-employed					
Yes	3,621	1,204	31.6%	1.25 (1.09–1.45)	Not applicable
No	2,271	655	26.9%	Reference	
Old age/disability pension					
Yes	2,067	600	27.1%	0.83 (0.72–0.95)	Not applicable
No	3,825	1,259	31.0%	Reference	
Unemployed					
Yes	350	101	24.9%	0.77 (0.58–1.02)	Not applicable
No	5,542	1,758	30.2%	Reference	
Work during restrictions					
Yes	3,542	1,174	31.6%	1.24 (1.08–1.43)	1.40 (1.16–1.69)
No	2,350	685	27.0%	Reference	
Mainly remote work (during restrictions)					
Yes	684	165	21.1%	0.60 (0.48–0.75)	0.61 (0.48–0.77)
No	5,208	1,694	30.8%	Reference	
Ways of working during restrictions (if employed or self-employed)					
Mainly remote work	684	165	21.1%	Reference	Not applicable
Mixture of working remotely and in a work setting	903	284	28.8%	1.51 (1.16–1.98)	
In a work setting with high degree of physical proximity	1,200	436	35.4%	2.05 (1.59–2.63)	
In a work setting with limited degree of physical proximity	755	289	35.1%	2.02 (1.53–2.66)	
Did not work due to restrictions	79	30	31.4%	1.71 (0.98–2.99)	
Other factors					
Contact with a known COVID-19 case					
Yes	1,869	820	42.4%	2.24 (1.95–2.58)	2.19 (1.89–2.55)
No	4,023	1,039	24.8%	Reference	
Received at least one dose of vaccine against COVID-19					
Yes	2,173	456	18.6%	Reference	
No	3,719	1,403	34.7%	2.33 (1.99–2.72)	2.72 (2.24–3.30)

CI: confidence interval; AOR: adjusted odds ratio; OR: odds ratio; SARS-CoV-2: severe acute respiratory syndrome coronavirus 2.

^a^ Weighted seroprevalence estimates calculated using the final weights, $w_{k}$ (see the definition in the Supplement).

^b^ The OR was adjusted in the final multivariable logistic model.

In a subset of participants who worked during pandemic, the highest stratified seroprevalence was seen in respondents who since March 2020 had not worked remotely (35.2%; 95% CI: 32.8–37.8), regardless of where they lived (p = 0.350). This group of respondents had twice the odds (AOR = 2.07; 95% CI: 1.53–2.72) of being seropositive compared with respondents who worked mainly from home. A mixture of work type, i.e. working remotely and in a work setting, was also associated with higher seropositivity (AOR = 1.41; 95% CI: 1.02–1.94) when compared with remote work only.

## Discussion

We report results of the Polish countrywide seroepidemiological survey on SARS-CoV-2 seropositivity based on a random sample of adults with a focus on infection-induced immunity. We found that by 14 May 2021, when the third wave of pandemic began in Poland, nearly one-third (29.8%) of the adult population had already developed antibodies following exposure to SARS-CoV-2. This percentage was unexpectedly high, given that the population recently infected during the third wave had not yet developed antibodies. Two studies comparable with our survey in terms of study protocol and timing reported lower estimates of infection-induced immunity in Belgium (17.0%) and Portugal (13.1%) among the unvaccinated [15,16].

In our study, prevalence of past SARS-CoV-2 infection in rural areas and small towns (32.2%; 95% CI: 30.2–34.4) was clearly higher than in mid-size and large cities (26.6%; 95% CI: 24.9–28.3). Overall, eastern Poland, with a higher proportion of rural population than the rest of the country (51% vs 37% as in June 2020) [17] was most affected. More importantly, the rurality effect was consistent across the country, with regional seroprevalence estimates being positively correlated with the proportion of the population living in rural and small-town areas.

A strongly differentiated and time-varying urban–rural gradient was observed primarily in the United States. There is evidence that intensity of SARS-CoV-2 transmission shifted from urban to vulnerable rural areas in 2020, although densely populated metropolitan areas were affected earlier [7]. According to reports from Europe, rural areas were not at higher risk of COVID-19 spread and, in fact, often reported lower infection rates [2]. In addition, research worldwide consistently shows that areas with a higher share of elderly or vulnerable people, as well as poor access to healthcare, are more vulnerable to the epidemic [2,18].

Although no single risk factor has been identified to explain the vulnerability of rural communities in Poland, we found significant differences in social structure and housing between these two settings. Rural participants lived in larger households with people > 60 years and a child under the age of 18 years. Indeed, 17.4% of the rural population in Poland (vs with 7.1% in cities) live in large, at least three-generational households [19]. Multigenerational housing is a risk factor independent of overcrowding, according to research [20], as it is likely to include someone with an underlying condition [21] and someone who cannot easily avoid exposure for SARS-CoV-2, such as a school-aged child or a working-age adult [22].

Our hypothesis is further corroborated by numerous population studies, which have also shown that subpopulations with a higher share of multigenerational households were disproportionally affected by COVID-19 in the United Kingdom [22]. Even after controlling for sociodemographic and underlying health issues, this factor was associated with a higher incidence of COVID-19 and/or mortality. Household density was considered an alternative hypothesis. However, the average surface of the dwelling per occupant is comparable in rural and urban areas of Poland (31.4 m^2^ vs 29.8 m^2^) [17]. Further, work-related exposures were clearly implicated but were accounted for in the multivariable model.

Healthcare access disparities between rural and urban areas probably exacerbated the observed gradient. Rural residents in Poland seek outpatient treatment at a lower rate than city dwellers (3 vs 12 times annually per resident in 2021) [23], which limits testing and contact-tracing services and contributes to an undercounting of cases [24]. Based on international evidence, lower participation in COVID-19-related preventive health behaviours and lower compliance with recommended or mandated public health measures is to be expected among rural residents [25,26]. It should also be noted that Poland imposed broad physical distancing measures in the early months of COVID-19, which probably slowed down the spread of SARS-CoV-2 in more densely populated urban areas and exacerbated the observed trends [8,27].

In addition, women over 60 years in rural areas and small cities had an unexpectedly high seroprevalence. While this finding requires confirmation, we note that among older women, COVID-19 hospitalisation rates were higher in rural areas despite being lower overall than among males [28]. Studies show that women in their working years may have higher infection rates than men, whereas among elderly people, men have usually higher infection rates overall and in particular higher fatality rates [29].

Finally, our survey confirmed the heavy burden of COVID-19 in Poland. By extrapolating our seroprevalence estimates to the population at risk, we conservatively estimate that by the end of the survey, a total of 9,118,768 (95% CI: 8,695,186–9,553,982) had been infected with SARS-CoV-2. The cumulative reporting fraction of COVID-19 infections to national surveillance for the entire country was 27.4% (95% CI: 26.2–28.7). These estimates fall within the predicted detection ratio projected for Poland in other research [10]. Regional estimates varied from 14.5% (95% CI: 12.2–17.8) to 35.7% (95% CI: 28.3–45.8) and were negatively correlated with the proportion of rural and small-town residents (R = −0.589; p = 0.017; n = 16), supporting the hypothesis of lower detection and reporting rates in rural areas.

Based on our findings, we would advise that public health messages and COVID-19 prevention strategies be tailored to local communities and regional needs.

Our study has several major strengths. This was the first nationwide seroepidemiological survey of SARS-CoV-2 infections based on a random sample of adults in Poland. The recruitment process and weighting adjustments ensured that the sample was representative of the target population, and the timing of the study in relation to the epidemic timeline was optimal for the assessment of infection-induced immunity. The survey was completed at the beginning of Poland's mass immunisation efforts, thus capturing the landscape of the SARS-CoV-2 epidemic and showing important regional differences despite the general wide spread of the epidemic in the whole country.

Our findings may have been hampered by a high non-participation rate (76.6%) in anti-SARS-CoV-2 antibody testing. All seroprevalence estimates have been adjusted (propensity score) for relevant factors associated with the participation. However, we acknowledge that some residual bias might still exist if there were additional factors associated with seropositivity but not ascertained in the questionnaire, such as education, income, availability of or proximity to the collection site, etc. Self-reported vaccination status was also considered a possible limitation, but it is unlikely that this was falsely reported so early in the vaccination programme. We note that the observed non-response was not influenced by SARS-CoV-2 risk depending on place of residence, which may have compromised the validity of the comparison between urban and rural areas. We also acknowledge the CATI survey's low response rate (11.5%). Phone surveys have recently suffered from decreased response rates due to changes in people's overall attitudes towards surveys, which are now frequently far below 10% [30]. We used weighting adjustments based on the main sociodemographic characteristics of the telephone survey respondents to bring the original sample composition closer to the reference population, but given the response rate, we cannot rule out that certain relevant groups of the population were under- or over-represented.

## Conclusions

Our results demonstrate regional vulnerability to COVID-19 in Poland. This study suggests that residents of rural communities and small cities in Poland are at increased risk of SARS-CoV-2. It highlights some differences in demographic and household structures which are likely to contribute to these disparities. Focused research on these groups would help determine which control measures would benefit them the most. 

## Supplementary Data

589000 Supplement
