# Supplementary material for: Reversed urban–rural gradient in COVID-19 seroprevalence and related factors in a nationally representative survey, Poland, 29 March to 14 May 2021
Source: Euro Surveill. 2023 Aug 31;28(35):2200745. doi: 10.2807/1560-7917.ES.2023.28.35.2200745 (PMC10472750; doi:10.2807/1560-7917.ES.2023.28.35.2200745)
Supplement: Supplement [file 22-00745_CZERWINSKI_Supplement.pdf]

Supplement

This supplementary material is hosted by Eurosurveillance as supporting information alongside the article Reversed urban-rural gradient in COVID-19 seroprevalence and related factors in a nationally representative survey in Poland: 29 March – 14 May, 2021, on behalf of the authors, who remain responsible for the accuracy and appropriateness of the content. The same standards for ethics, copyright, attributions and permissions as for the article apply. Supplements are not edited by Eurosurveillance and the journal is not responsible for the maintenance of any links or email addresses provided therein.

Supplementary tables and figures

Supplementary **figure S1**. Study enrollment flow chart and studied sample in OBSER-CO cross-sectional survey in Poland: 29 March - 14 May, 2021 3

Supplementary **table S1**. Sociodemographic characteristics of the final sample of CATI respondents and serosurvey participants compared to general (Polish) population in OBSER-CO cross-sectional survey in Poland: 29 March - 14 May, 2021 4

Supplementary **table S2**. Sociodemographic characteristics of the final sample of serosurvey participants compared to CATI respondents in the OBSER-CO cross-sectional survey in Poland: 29 March - 14 May, 2021.. 5

Supplementary **table S3**. Characteristics of serosurvey participants from rural areas/small cities (population under 50,000) compared to residents of mid-sized/large cities (population of 50,000 or more) in the OBSER-CO cross-sectional survey in Poland: 29 March - 14 May, 2021 6

Supplementary **table S4**. Presentation for the serological test among participants of CATI survey, by demographic and other characteristics 11

Supplementary **table S5**. Final multivariable logistic regression model predicting the probability of presenting for the serological test among the CATI respondents 14

Supplementary **table S6**. Final multivariable logistic regression model used for estimating adjusted ORs 18

Supplementary **table S7**. Replication of main results from Tables 1-3 without Weights, wk. 20

Supplementary **table S8**. Replication of main results from Tables 1-3 with borderline laboratory results 22

Supplementary descriptions of methodology

Statistical weights development methodology ... 7
Concept ... 7
Development of stage 1 weight, $w1$ ... 8
Development of stage 2 weight ... 10
Multivariable Regression Analysis ... 16
Impact of weights on estimated seroprevalence ... 19
Impact of exclusion of laboratory borderline results on rural-urban gradient ... 21
References ... 23

Supplementary **figure S1**. Study enrollment flow chart and studied sample in OBSER-CO cross-sectional survey in Poland: 29 March - 14 May, 2021

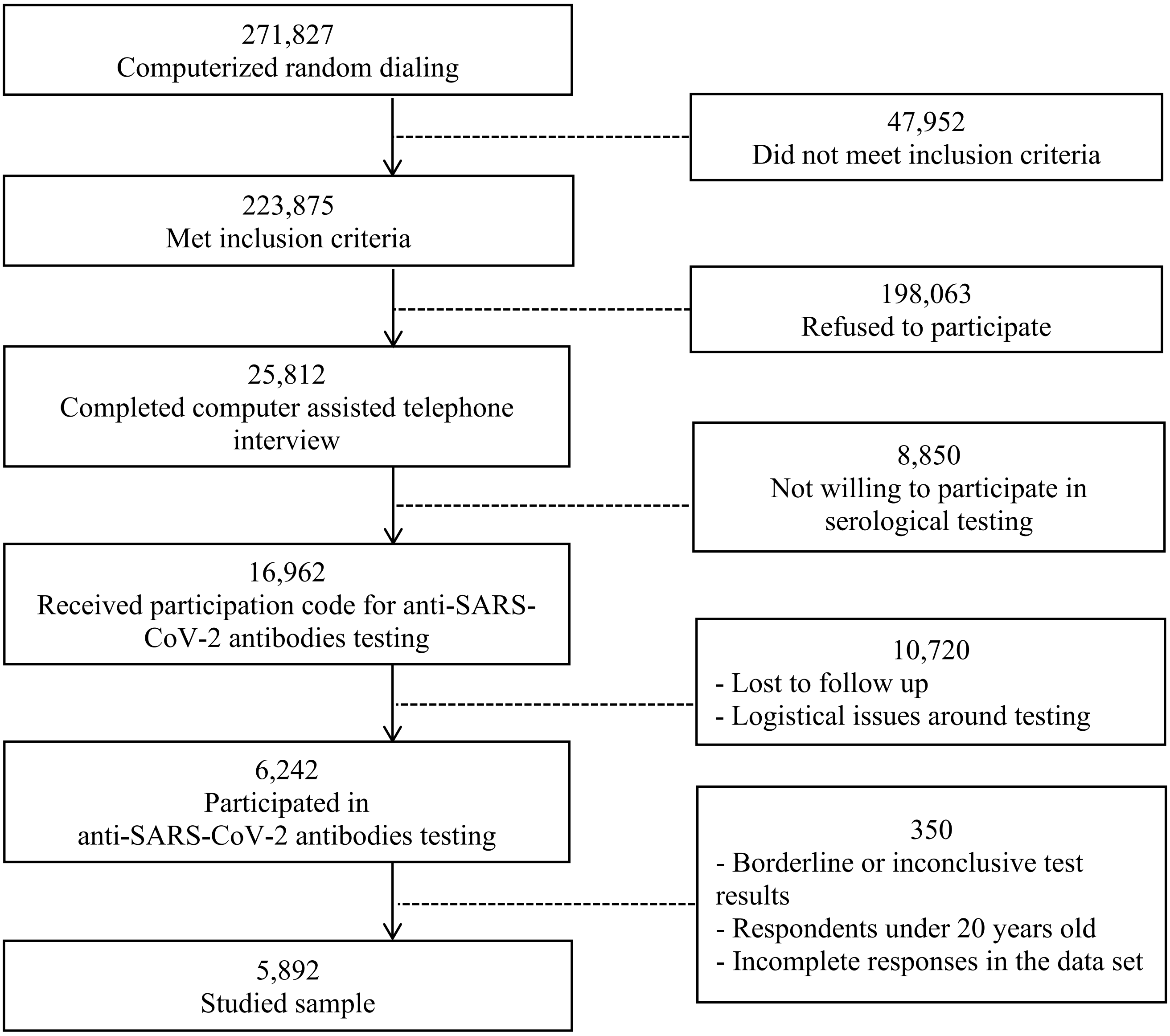

Supplementary **table S1**. Sociodemographic characteristics of the final sample of CATI respondents and serosurvey participants compared to general (Polish) population in OBSER-CO cross-sectional survey in Poland: 29 March - 14 May, 2021

Characteristics	Polish population		CATI respondents		Serosurvey participants			p value[2]
	N	crude %	N	crude %	N	crude %	adj. %[1]	
Age group (years)								**0.1521**
20-39	10 418 299	**34.1%**	6 433	25.5%	1 392	23.6%	**32.8%**	
40-59	10 373 837	**33.9%**	8 302	32.9%	2 163	36.7%	**35.0%**	
60-69	5 185 843	**17.0%**	5 694	22.6%	1 424	24.2%	**17.3%**	
70+	4 614 390	**15.1%**	4 773	18.9%	913	15.5%	**14.9%**	
Sex								**0.0084**
females	16 028 016	**52.4%**	13 529	53.7%	3 455	58.6%	**54.1%**	
males	14 564 353	**47.6%**	11 673	46.3%	2 437	41.4%	**45.9%**	
Place of residence								**0.7980**
rural areas	12 032 388	**39.3%**	7 099	28.2%	1 406	23.9%	**39.5%**	
urban areas	18 559 981	**60.7%**	18 103	71.8%	4 486	76.1%	**60.5%**	
Region								**0.8781**
1. Lower Silesian	2 343 528	**7.7%**	1 796	7.1%	400	6.8%	**7.8%**	
2. Kuyavian-Pomeranian	1 649 892	**5.4%**	1 402	5.6%	226	3.8%	**5.4%**	
3. Lublin	1 685 507	**5.5%**	1 684	6.7%	338	5.7%	**5.4%**	
4. Lubusz	805 941	**2.6%**	760	3.0%	181	3.1%	**2.7%**	
5. Łódź	1 978 274	**6.5%**	1 670	6.6%	399	6.8%	**6.8%**	
6. Lesser Poland	2 691 170	**8.8%**	1 837	7.3%	557	9.5%	**8.3%**	
7. Masovian	4 275 835	**14.0%**	3 712	14.7%	900	15.3%	**14.1%**	
8. Opole	803 139	**2.6%**	897	3.6%	164	2.8%	**2.5%**	
9. Subcarpathian	1 692 792	**5.5%**	1 526	6.1%	288	4.9%	**6.0%**	
10. Podlaskie	945 286	**3.1%**	1 000	4.0%	240	4.1%	**2.9%**	
11. Pomeranian	1 835 873	**6.0%**	1 535	6.1%	455	7.7%	**6.4%**	
12. Silesian	3 642 082	**11.9%**	1 957	7.8%	487	8.3%	**11.9%**	
13. Holy Cross	999 835	**3.3%**	1 083	4.3%	257	4.4%	**3.2%**	
14. Warmian–Masurian	1 130 158	**3.7%**	1 121	4.4%	236	4.0%	**3.6%**	
15. Greater Poland	2 746 826	**9.0%**	1 822	7.2%	475	8.1%	**8.8%**	
16. West Pomeranian	1 366 231	**4.5%**	1 400	5.6%	289	4.9%	**4.3%**	
COVID-19 vaccination status								**0.0487**
at least one dose	8 972 682	**29.3%**	9 317	37.0%	2 173	36.9%	**30.5%**	
not vaccinated	21 619 687	**70.7%**	15 885	63.0%	3 719	63.1%	**69.5%**	

[1] weighted distribution calculated using the final weights, w_k

[2] for Chi-Square difference between the weighted distribution and the crude % of Polish population

Supplementary **table S2**. Sociodemographic characteristics of the final sample of serosurvey participants compared to CATI respondents in the OBSER-CO cross-sectional survey in Poland: 29 March - 14 May, 2021

Age group (in years)	Characteristics	CATI respondents		Serosurvey participants			p value[3]
		N	adj %[1]	N	crude %	adj %[2]	
all		25 202	**100.0%**	5 892	100.0%	**100.0%**	
	Household size (persons in a household)						
20-39	1 - 2	2 367	**31.3%**	517	37.1%	**31.3%**	**0.7928**
	3 - 4	3 055	**49.1%**	697	50.1%	**49.8%**	
	5 or more	1 011	**19.6%**	178	12.8%	**18.8%**	
40-59	1 - 2	2 951	**34.3%**	739	34.2%	**31.5%**	**0.0340**
	3 - 4	4 127	**49.7%**	1 133	52.4%	**52.5%**	
	5 or more	1 224	**16.0%**	291	13.5%	**16.0%**	
60+	1 - 2	8 064	**75.6%**	1 865	79.8%	**77.0%**	**0.3320**
	3 - 4	1 731	**16.9%**	350	15.0%	**15.7%**	
	5 or more	672	**7.5%**	122	5.2%	**7.3%**	
all	1 - 2	13 382	**46.5%**	3 121	53.0%	**46.1%**	**0.4813**
	3 - 4	8 913	**39.0%**	2 180	37.0%	**39.8%**	
	5 or more	2 907	**14.5%**	591	10.0%	**14.1%**	
	Cohabiting with a child younger than 18						
20-39	none	3 329	**48.4%**	635	45.6%	**43.3%**	**0.0006**
	1 or more	3 104	**51.6%**	757	54.4%	**56.7%**	
40-59	none	4 829	**57.7%**	1 202	55.6%	**58.7%**	**0.3931**
	1 or more	3 473	**42.3%**	961	44.4%	**41.3%**	
60+	none	9 718	**92.1%**	2 193	93.8%	**92.5%**	**0.5017**
	1 or more	749	**7.9%**	144	6.2%	**7.5%**	
all	none	17 876	**65.5%**	4 030	68.4%	**64.5%**	**0.1447**
	1 or more	7 326	**34.5%**	1 862	31.6%	**35.5%**	
	Source of income						
	employed / self-employed	14 203	**61.7%**	3 621	61.5%	**62.5%**	**0.2346**
	old age/disability pension	9 518	**30.1%**	2 067	35.1%	**30.0%**	**0.9006**
	unemployed	1 775	**8.4%**	350	5.9%	**8.3%**	**0.8100**
all	Ways of working (if in employment)						**0.4991**
	mainly remotely	2 424	**16.5%**	684	18.9%	**16.1%**	
	mixture - remotely and in a setting	2 971	**20.1%**	903	24.9%	**20.5%**	
	in a work setting	8 455	**60.7%**	1 955	54.0%	**61.1%**	
	did not work due to restrictions	353	**2.7%**	79	2.2%	**2.3%**	
	Received at least 1 dose of COVID Vaccine						
20-39		678	**11.2%**	153	11.0%	**11.1%**	**0.9427**
40-59		1921	**21.4%**	504	23.3%	**20.7%**	**0.4910**
60+		6718	**57.0%**	1 516	64.9%	**61.0%**	**0.0004**
all		9 317	**29.3%**	2 173	36.9%	**30.5%**	**0.0690**
all	Contact with confirmed COVID case						**0.3718**
	no contact	18 644	**72.2%**	4 023	68.3%	**71.4%**	
	contact at one time	2 557	**10.5%**	752	12.8%	**11.0%**	
	casual contact	4 001	**17.2%**	1 117	19.0%	**17.6%**	

[1] weighted distribution calculated using w^1 weights

[2] weighted distribution calculated using the final weights, w_k

[3] for Chi-Square difference between the weighted distributions of Serosurvey and CATI participants

Supplementary **table S3**. Characteristics of serosurvey participants from rural areas/small cities (population under 50,000) compared to residents of mid-sized/large cities (population of 50,000 or more) in the OBSER-CO cross-sectional survey in Poland: 29 March - 14 May, 2021

Age group (in years)	Characteristics	N	Rural/urban areas (pop. under 50,000) n	adj %[1]	Urban areas (pop. of 50,000 or more) n	adj %[1]	p value
all		5 892	2 803	100.0%	3 089	100.0%	
	Household size (persons in a household)						
20-39	1 - 2	517	113	18.4%	404	47.7%	<0.001
	3 - 4	697	322	54.5%	375	43.8%	
	5 or more	178	120	27.1%	58	8.4%	
40-59	1 - 2	739	314	25.5%	425	40.8%	<0.001
	3 - 4	1 133	593	55.2%	540	48.4%	
	5 or more	291	183	19.4%	108	10.8%	
60+	1 - 2	1 865	852	70.3%	1 013	85.2%	<0.001
	3 - 4	350	214	18.5%	136	12.3%	
	5 or more	122	92	11.2%	30	2.5%	
all	1 - 2	3 121	1 279	37.1%	1 842	58.1%	<0.001
	3 - 4	2 180	1 129	43.6%	1 051	34.7%	
	5 or more	591	395	19.3%	196	7.2%	
	Cohabiting with a child younger than 18						
20-39	none	635	180	33.4%	455	55.9%	<0.001
	1 or more	757	375	66.6%	382	44.1%	
40-59	none	1202	610	58.9%	592	58.4%	0.836
	1 or more	961	480	41.1%	481	41.6%	
60+	none	2 193	1 051	89.1%	1 142	96.8%	<0.001
	1 or more	144	107	10.9%	37	3.2%	
all	none	4 030	1 841	60.1%	2 189	70.5%	<0.001
	1 or more	1 862	962	39.9%	900	29.5%	
	Source of income						
	employed / self-employed	3 621	1 626	61.1%	1 995	64.5%	0.027
	old age/disability pension	2 067	1 052	29.9%	1 015	30.2%	0.813
	unemployed	350	192	9.7%	158	6.4%	<0.001
all	Ways of working (if in employment)						<0.001
	mainly remotely	684	217	12.3%	467	21.0%	
	mixture - remotely and in a work setting	903	354	17.4%	549	24.5%	
	in a work setting	1 955	1 019	68.1%	936	52.1%	
	did not work due to restrictions	79	36	2.2%	43	2.4%	
	Received at least 1 dose of COVID Vaccine						
20-39		153	67	12.4%	86	9.4%	0.132
40-59		504	253	21.2%	251	20.0%	0.540
60+		1 516	730	60.1%	786	62.2%	0.405
all		2 173	1 050	30.5%	1 123	30.6%	0.896
all	Contact with confirmed COVID case						0.002
	no contact	4 023	1 883	70.2%	2 140	73.1%	
	contact at one time	752	349	10.6%	403	11.5%	
	casual contact	1 117	571	19.2%	546	15.4%	

[1] weighted % estimates calculated using the final weights, w_k

Statistical weights development methodology

Concept

Due to the design of the recruitment process we employed a two-stage adjustment to our SARS-CoV-2 seroprevalence estimates to account for non-response at the level of telephone interview and at the level of presentation for the test and receiving the valid test result. The method is an extension of Horvitz–Thompson (HT) estimator for population parameters with non-response weighting, which was shown to be an unbiased although not efficient estimator [1].
We assume non-informative non-response, that is that given the covariates, the probability of coming for the test does not depend on the test outcome and use an approach discussed by Little et al [2]. The paper considers the sampling weights resulting from the sampling design in combination with the non-response weights to account for the lack of participation of the sampled individuals. In our case, as the probability of participation in the telephone survey (CATI, computer assisted telephone interview) is related to a mixture of the design (phone number coverage and availability) factors and the lack of consent for the interview, we call it simply the stage 1 weight, w^1. Secondly, the inverse of the probability of presenting for a laboratory test and receiving valid test results among those who took part in the telephone survey was denoted as stage 2 weight, w^2.
The weighted estimate of population mean is given by the formula:

$$\hat{Y} = \sum_j \sum_k \widehat{P_{jk}} * \widehat{Y_{jk}}$$

Where $\widehat{P_{jk}} = w_j^1 * w_{jk}^2 = \left(N_{j+}/N\right) * \left(n_{jk}/n_{j+}\right)$, is the estimated proportion of the population in demographic stratum j, who in addition are in the individual characteristic cell k. N_{j+} is the size of the demographic stratum in the total population of size N; n_{jk}- number of individuals in the sample recruited in the first step (CATI interview) who fall into the demographic stratum j and have characteristic k, measured for the CATI respondents, of all CATI respondents in the demographic stratum j.
It has been suggested that in the case of two stage weighting the resulting estimates are approximately unbiased if the stage 1 weight is constant for the stratum defined to develop the stage 2 weight [2]. The demographic variables used for stage 1 were therefore always considered in the development of stage 2 weights.

Development of stage 1 weight, w^1

Stage one weight was used to adjust the distribution of our sample of CATI telephone survey respondents to the structure of the reference Polish population as of June 2020, as provided by the National Statistical Office of Poland.
We initially considered the following factors for stage 1 weighting:
- administrative region (voivodeship) – there are 16 voivodeships in Poland
- sex (male; female)
- age group (20-39; 40-59; 60-69; ≥70 years)
- type of residence (rural; urban).
- COVID-19 vaccination status (at least one dose; not vaccinated)

The COVID-19 vaccination status was added as a possible indicator of interest in or fear of the pandemic that could impact the likelihood of participating in the study. The study was conducted 3 - 4 months after the vaccine became available in Poland, first to older age groups and vulnerable populations, and approximately at the time of the study – to the whole adult population. Vaccination coverage in the general population was available from the eHealth Centre, thanks to an electronic system newly developed to track COVID-19 vaccination coverage [3].

The stratification above resulted in 16*2*4*2*2 = 512 strata. In order to ensure that the resulting strata were not empty, we analysed the distribution of the number of study participants in each stratum.

Supplementary figure S2. Distribution of strata by the number of cases in each stratum.
A – all strata, B – only strata with <=5 cases

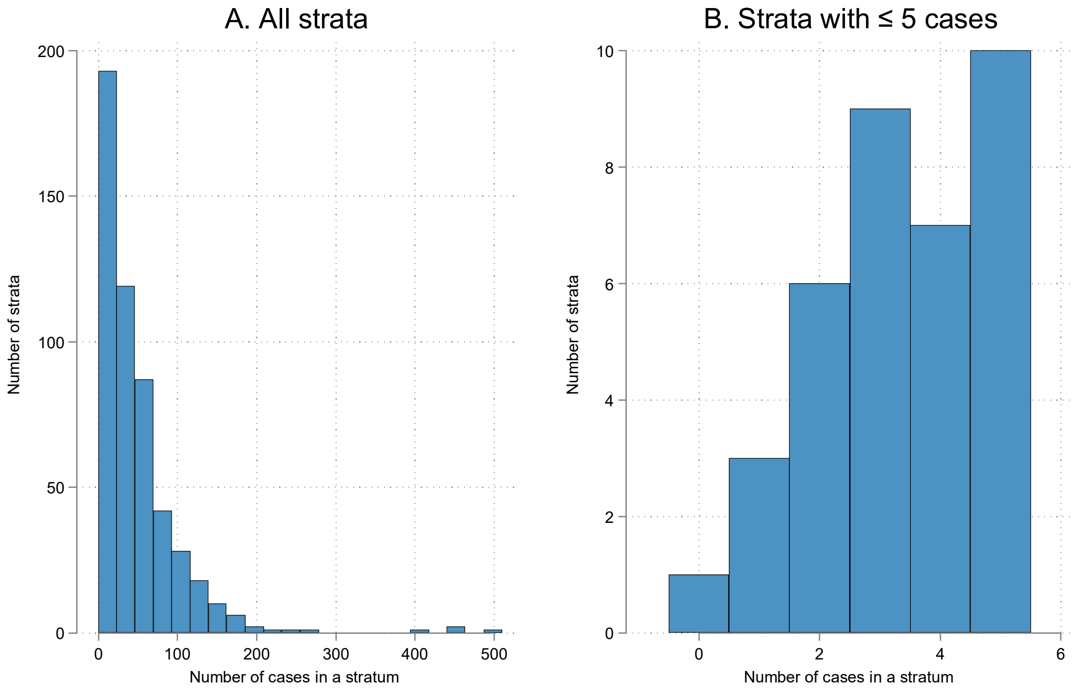

The stratification resulted in one empty stratum and 25 strata with the count of 1- 4, which were considered too small for weighting. In order to avoid empty weighting strata, we arbitrarily

decided to combine males and females, and the proportion of men and women was comparable among the CATI respondents and the general population (Supplementary **table S1**).
The stratum weights w^1 were determined by dividing the proportions of the strata in the reference population by the proportion of the corresponding cells in the stratified sample. They were normalised so that the sum of the weights was equal to the total sample size of CATI respondents.
Large weights were not removed at this stage. The w^1 ranged from 0.21 to 35.6. However, the 99% percentile was 5.4, indicating that the vast majority was within acceptable limits.

Development of stage 2 weight

The CATI respondents filled in an interview targeting COVID-19 risk factors. At the same time the interview allowed for a more detailed analysis of the non-response at the level of presenting for the serological test and the design of the appropriate adjustment. Given that if all the variables from stage 1 weighting were taken together with risk factor variables the resulting cells would be either very small or empty, we applied a logistic regression model predicting presentation for testing among the CATI interview respondents. The stage 1 variables were also included in the model to maintain the possibility of generalizing the final results to the population [2,4].

The factors initially considered to predict the presentation for a serological test included:

- The demographic factors (gender, age group, residence type, voivodship) as in the w^1 definition
- Vaccination status (Not vaccinated, Vaccinated with one does, Vaccinated with 2 doses)
- Having experienced any symptoms from the list: fever, cough, shortness of breath, loss of smell and/or taste, sore throat, running nose, muscle or joint aches, fatigue, headache, stomach ache, nausea/vomiting, diarrhoea, rash, conjunctivitis, chills, loss of appetite, bloody nose, confusion, other neurological symptoms
- Having experienced any of the typical COVID-19 symptoms: fever, cough, shortness of breath, loss of smell and/or taste
- Having close relatives and friends (who were not members of the household) met daily
- Employment status (Employed; Unemployed; On pension or retired; Student or in training
- Type of work (remote or mainly remote; other)
- Any contact with a confirmed case
- Prior diagnosis of COVID-19
- Being on sick leave due to COVID-19 compatible symptoms
- Hospitalized due to COVID-19 or respiratory tract infection
- Number of household members (1; 2; 3; 4; 5 or more)
- Number of children under 18 living in the same household (1; 2; 3; 4; 5 or more)
- Participation in regular meetings outside of work (e.g., sports or art clubs)
- Participation in an organized trip or mission outside of the city of residence
- Participation in a private gathering such as a wedding

Recall period used for the questions: since March 2020 (approximately 13 months).

Supplementary **table S4**. Presentation for the serological test among participants of CATI survey, by demographic and other characteristics

Factors:	Outcome: Presentation for serological test			
	No		Yes	
	N	Row %	N	Row %
Residence type				
Rural (n=7,099)	5617	79.1	1482	20.9
Urban (n=18,103)	13376	73.9	4727	26.1
Total (n=25,202)	18993	75.4	6209	24.6
Pearson chi2(1) = 75.2811 Pr = 0.000				
Age group				
20-39 (n=6,433)	4954	77.0	1479	23.0
40-59 (n=8,302)	6026	72.6	2276	27.4
60-69 (n=5,694)	4193	73.6	1501	26.4
≥70 years (n=4,773)	3820	80.0	953	20.0
Total (n=25,202)	18993	75.4	6209	24.6
Pearson chi2(3) = 109.0892 Pr = 0.000				
Sex				
Females (n=13,529)	9884	73.1	3645	26.9
Males (n=11,673)	9109	78.0	2564	22.0
Total (n=25,202)	18993	75.4	6209	24.6
Pearson chi2(1) = 83.5962 Pr = 0.000				
Region				
1.Lower Silesian (n=1,796)	1372	76.4	424	23.6
2.Kuyavian-Pomeranian (n=1,402)	1161	82.8	241	17.2
3.Lublin (n=1,684)	1332	79.1	352	20.9
4.Lubusz (n=760)	569	74.9	191	25.1
5.Łódź (n=1,670)	1255	75.1	415	24.9
6.Lesser Poland (n=1,837)	1244	67.7	593	32.3
7.Masovian (n=3,712)	2768	74.6	944	25.4
8.Opole (n=897)	726	80.9	171	19.1
9.Subcarpathian (n=1,526)	1222	80.1	304	19.9
10.Polaskie (n=1,000)	749	74.9	251	25.1
11.Pomeranian (n=1,535)	1062	69.2	473	30.8
12.Silesian (n=1,957)	1441	73.6	516	26.4
13.Holy Cross (n=1,083)	813	75.1	270	24.9
14.Warmian–Masurian (n=1,121)	866	77.3	255	22.7
15.Greater Poland (n=1,822)	1323	72.6	499	27.4
16.West Pomeranian (n=1,400)	1090	77.9	310	22.1
Total (n=25,202)	18993	75.4	6209	24.6
Pearson chi2(15) = 197.1825 Pr = 0.000				
Prior diagnosis of COVID-19 since March 2020				
Yes (n=3,514)	2426	69.0	1088	31.0
No (n=21,688)	16567	76.4	5121	23.6
Total (n=25,202)	18993	75.4	6209	24.6
Pearson chi2(1) = 87.9800 Pr = 0.000				
Contact with a confirmed case since March 2020				
No (n=18,644)	14436	77.4	4208	22.6
Yes (n=6,558)	4557	69.5	2001	30.5
Total (n=25,202)	18993	75.4	6209	24.6
Pearson chi2(1) = 164.8161 Pr = 0.000				
Symptoms compatible with COVID-19 since March 2020				
No (n=8,885)	7244	81.5	1641	18.5
Not typical (n=6,803)	5075	74.6	1728	25.4
Typical (n=9,514)	6674	70.1	2840	29.9
Total (n=25,202)	18993	75.4	6209	24.6
Pearson chi2(2) = 323.4603 Pr = 0.000				
Being on sick leave due to symptoms compatible with COVID-19				
Yes (n=3,175)	2183	68.8	992	31.2
No (n=22,027)	16810	76.3	5217	23.7
Total (n=25,202)	18993	75.4	6209	24.6
Pearson chi2(1) = 85.4098 Pr = 0.000				

Factors:	Outcome: Presentation for serological test			
	No		Yes	
	N	Row %	N	Row %
Hospitalized due to COVID-19 or respiratory tract infection				
Yes (n=322)	242	75.2	80	24.8
No (n=24,880)	18751	75.4	6129	24.6
Total (n=25,202)	18993	75.4	6209	24.6
Pearson chi2(1) = 0.0076 Pr = 0.931				
Type of work since March 2020				
Other situation (n=19,807)	15283	77.2	4524	22.8
Remote of mainly remote (n=5,395)	3710	68.8	1685	31.2
Total (n=25,202)	18993	75.4	6209	24.6
Pearson chi2(1) = 160.8359 Pr = 0.000				
Employment status				
Employed (n=11,590)	8427	72.7	3163	27.3
Unemployed (n=11,837)	9154	77.3	2683	22.7
Other situation (n=1,775)	1412	79.5	363	20.5
Total (n=25,202)	18993	75.4	6209	24.6
Pearson chi2(2) = 85.4758 Pr = 0.000				
Student or in training				
No (n=24,361)	18325	75.2	6036	24.8
Yes (n=841)	668	79.4	173	20.6
Total (n=25,202)	18993	75.4	6209	24.6
Pearson chi2(1) = 7.7476 Pr = 0.005				
On pension or retired				
No (n=15,684)	11651	74.3	4033	25.7
Yes (n=9,518)	7342	77.1	2176	22.9
Total (n=25,202)	18993	75.4	6209	24.6
Pearson chi2(1) = 25.9519 Pr = 0.000				
Number of household members				
1 (n=4,682)	3684	78.7	998	21.3
2 (n=8,700)	6423	73.8	2277	26.2
3 (n=4,687)	3532	75.4	1155	24.6
4 (n=4,226)	3078	72.8	1148	27.2
5 and more (n=2,907)	2276	78.3	631	21.7
Total (n=25,202)	18993	75.4	6209	24.6
Pearson chi2(4) = 66.8594 Pr = 0.000				
Number of children < 18 in the household				
None (n=17,876)	13640	76.3	4236	23.7
1 (n=3,457)	2561	74.1	896	25.9
2 (n=3,008)	2150	71.5	858	28.5
3 (n=659)	485	73.6	174	26.4
4 (n=134)	106	79.1	28	20.9
5 and more (n=68)	51	75.0	17	25.0
Total (n=25,202)	18993	75.4	6209	24.6
Pearson chi2(5) = 38.1713 Pr = 0.000				
Participation in a private gathering such as a wedding since March 2020				
Yes (n=7,784)	5598	71.9	2186	28.1
No (n=17,418)	13395	76.9	4023	23.1
Total (n=25,202)	18993	75.4	6209	24.6
Pearson chi2(1) = 72.0450 Pr = 0.000				
Having close relatives and/or friends (who were not members of the household) met daily				
Yes (n=20,702)	15382	74.3	5320	25.7
No (n=4,500)	3611	80.2	889	19.8
Total (n=25,202)	18993	75.4	6209	24.6
Pearson chi2(1) = 70.3032 Pr = 0.000				
Participation in regular meetings outside of work (e.g. sports or art clubs)				
Yes (n=5,247)	3795	72.3	1452	27.7
No (n=19,955)	15198	76.2	4757	23.8
Total (n=25,202)	18993	75.4	6209	24.6
Pearson chi2(1) = 32.8972 Pr = 0.000				

Factors:	Outcome: Presentation for serological test			
	No		Yes	
	N	Row %	N	Row %
Participation in an organized trip or mission outside of the city of residence				
Yes (n=3,345)	2385	71.3	960	28.7
No (n=21,857)	16608	76.0	5249	24.0
Total (n=25,202)	18993	75.4	6209	24.6
Pearson chi2(1) = 34.2853 Pr = 0.000				
COVID-19 vaccination status				
One dose (n=5,877)	4359	74.2	1518	25.8
Two doses (n=3,440)	2621	76.2	819	23.8
Not vaccinated (n=15,885)	12013	75.6	3872	24.4
Total (n=25,202)	18993	75.4	6209	24.6
Pearson chi2(2) = 6.3605 Pr = 0.042				

All the above factors were initially entered into the logistic regression model. The backward selection procedure, with a retention level <0.1, removed two factors: Being on a pension or retired, Being on sick leave due to symptoms compatible with COVID-19.

Supplementary **table S5**. Final multivariable logistic regression model predicting the probability of presenting for the serological test among the CATI respondents

Factor:	Odds Ratio	Std. Err.	z	P>z	[95% Conf. Interval]
Residence type					
Rural	1.0	(base)			
Urban	1.3	0.0	7.93	0.000	1.2-1.4
Age group					
20-39	1.0	(base)			
40-59	1.4	0.1	8.64	0.000	1.3-1.6
60-69	1.6	0.1	8.40	0.000	1.4-1.8
70+	1.1	0.1	1.75	0.080	1.0-1.3
Sex					
Female	1.0	(base)			
Male	0.8	0.0	-9.34	0.000	0.7-0.8
Region					
1.Lower Silesian	1.0	(base)			
2.Kuyavian-Pomeranian	0.7	0.1	-4.35	0.000	0.6-0.8
3.Lublin	0.9	0.1	-1.23	0.220	0.8-1.1
4.Lubusz	1.1	0.1	0.63	0.529	0.9-1.3
5.Łódź	1.1	0.1	0.78	0.437	0.9-1.2
6.Lesser Poland	1.6	0.1	6.36	0.000	1.4-1.9
7.Masovian	1.0	0.1	0.51	0.609	0.9-1.2
8.Opole	0.8	0.1	-2.61	0.009	0.6-0.9
9.Subcarpathian	0.8	0.1	-1.99	0.047	0.7-1.0
10.Polaskie	1.1	0.1	1.16	0.245	0.9-1.3
11.Pomeranian	1.4	0.1	4.42	0.000	1.2-1.7
12.Silesian	1.1	0.1	1.70	0.089	1.0-1.3
13.Holy Cross	1.1	0.1	1.10	0.269	0.9-1.3
14.Warmian–Masurian	0.9	0.1	-0.62	0.538	0.8-1.1
15.Greater Poland	1.3	0.1	3.05	0.002	1.1-1.5
16.West Pomeranian	0.9	0.1	-0.93	0.352	0.8-1.1
Prior diagnosis of COVID-19 since March 2020					
Yes	1.0	(base)			
No	0.9	0.0	-2.07	0.038	0.8-1.0
Contact with a confirmed case since March 2020					
No	1.0	(base)			
Yes	1.2	0.0	6.37	0.000	1.2-1.3
Symptoms compatible with COVID-19 since March 2020					
No	1.0	(base)			
Not typical	1.4	0.1	9.16	0.000	1.3-1.6
Typical	1.8	0.1	14.33	0.000	1.6-1.9
Hospitalized due to COVID-19 or respiratory tract infection					
Yes	1.0	(base)			
No	1.3	0.2	1.86	0.063	1.0-1.7
Type of work since March 2020					
Other situation	1.0	(base)			
Remote or mainly remote	1.3	0.1	6.59	0.000	1.2-1.4
Employment status					
Employed	1.0	(base)			
Unemployed	0.9	0.0	-1.37	0.169	0.9-1.0
Other situation	0.9	0.1	-2.15	0.032	0.8-1.0
Student or in training					
No	1.0	(base)			
Yes	0.9	0.1	-1.45	0.148	0.7-1.0
Number of household members					
1	1.0	(base)			
2	1.3	0.1	5.94	0.000	1.2-1.4
3	1.1	0.1	1.53	0.126	1.0-1.2
4	1.1	0.1	1.20	0.231	1.0-1.2
5 and more	0.8	0.1	-2.14	0.032	0.7-1.0

Factor:	Odds Ratio	Std. Err.	z	P>z	[95% Conf. Interval]
Number of children < 18 in the household					
None	**1.0**	(base)			
1	**1.2**	0.1	2.77	0.006	1.0-1.3
2	**1.4**	0.1	4.88	0.000	1.2-1.6
3	**1.6**	0.2	4.00	0.000	1.3-2.0
4	**1.3**	0.3	1.15	0.251	0.8-2.0
5 and more	**1.5**	0.4	1.35	0.178	0.8-2.7
Participation in a private gathering such as a wedding since March 2020					
Yes	**1.0**	(base)			
No	**0.8**	0.0	-5.95	0.000	0.8-0.9
Having close relatives and/or friends (who were not members of the household) met daily					
Yes	**1.0**	(base)			
No	**0.8**	0.0	-6.04	0.000	0.7-0.8
Participation in regular meetings outside of work (e.g. sports or art clubs)					
Yes	**1.0**	(base)			
No	**0.9**	0.0	-3.76	0.000	0.8-0.9
Participation in an organized trip or mission outside of the city of residence					
Yes	**1.0**	(base)			
No	**0.9**	0.0	-2.98	0.003	0.8-1.0
COVID-19 vaccination status					
One dose	**1.0**	(base)			
Two doses	**1.1**	0.1	2.00	0.045	1.0-1.2
Not vaccinated	**0.9**	0.0	-3.03	0.002	0.8-1.0
_cons	**0.2**	0.0	-10.38	0.000	0.1-0.2

The model was used to predict the probability of success (presentation for a serological test) – the propensity score. The second stage weight for an individual, who presented for testing, w_k^2, was defined as the inverse of the propensity score.

Finally, the individual weight for the individual k, was defined as product of stage 1 and stage 2 weights normalized to obtain $\sum_{k=1\ldots T} w_k$ = T:

$$w_k = w_j^1 * w_k^2 * \frac{T}{\sum w_j^1 * w_k^2}$$

where the individual k belongs to demographic stratum j and the total sample of individuals tested is denoted as T.
These weights varied from 0.12 to 49.5, with the 1% percentile equal to 0.17 and 99% percentile equal to 7.0. We performed trimming of outlying weights to keep them within acceptable limits [5]. Upon trimming, weights varied from 0.15 to 8.81 (Mean = 1.00, Std. Dev. = 0 .72).

The data in supplementary **table S1** summarizes the distribution of demographic characteristics in the general adult population of Poland, the CATI respondents, and the serosurvey participants. Supplementary **table S2** compares the detailed sociodemographic characteristics of the final sample of serosurvey participants to those of the CATI respondents. The weighted distribution is provided (adj%) for the serorsurvey participants, calculated using the final weights, w_k. Upon weighting, the survey sample closely matches the desired population distribution.

Multivariable Regression Analysis

The multivariable logistic regression analysis was carried out on weighted data to examine effect modification, i.e., a two-way interaction between a place of residence and risk factors for COVID-19 infection with subject-matter importance. The initial model included all of the investigated main effects and all possible two-way interactions with place of residence (dichotomized as rural areas or small cities with populations of less than 50,000 vs. mid-sized or large cities with populations of 50,000 or more).

The following effects were assessed: region (voivodeship); age group (20-39, 40-59, 60-69, ≥70 years); sex (men, women); place of residence (categorized as rural areas/small cities under 50,000 population, mid-sized/large cities of 50,000 population and over); cohabiting with a child younger than 18 years old (yes, no); household size (number of people in a household treated as a continuous variable); income from old age/disability pension (yes, no); unemployed (yes, no); have worked during restrictions, i.e. since March 2020 (yes, no); have worked mainly remotely (yes, no); contact with a known COVID case (yes, no); and have received at least one dose of vaccine against COVID-19 (yes, no).

By applying a backward stepwise approach, we "forced" a complete assessment of all possible interactions (with a place of residence) that might be required in the final model. Then, in accordance with the model hierarchy (i.e., interactions must be removed before main effects), we removed all terms from the model that did not meet the significance criteria ($p<0.05$).
One of the difficulties encountered was the collinearity between household size and cohabiting with at least one child (the pairwise correlation coefficient = 0.6813), both important from a subject-matter standpoint. In this dataset, however, the latter predictor (cohabiting with at least one child) provided somewhat redundant information since this information was also contained in the first predictor (household size). The inclusion of household size in the multivariable model masked the significance of the second collinear variable (cohabiting with at least one child and its interaction term with place of residence). After consultation with COVID-19 data modelers, we have arbitrarily chosen to retain household size in the final model.

The following terms were eliminated in subsequent steps that did not meet the significance criteria: (1) interaction term between cohabiting with a child younger than 18 years old and place of residence; (2) cohabiting with a child younger than 18 years old; (3) interaction term between contact with a known COVID case and place of residence; (4) interaction term between have worked mainly remotely and place of residence; (5) interaction term between have received at least one dose of vaccine against COVID-19 and place of residence; (6) interaction term between income from old age/disability pension and place of residence; (7) income from old age/disability pension; (8) interaction term between unemployed and place of residence; (9) unemployed; (10) interaction term between have worked during restrictions and place of residence.

The final multivariable logistic regression model used to estimate adjusted odds ratios (ORs) is presented in Supplementary **table S6**. For main effects not included in interactions, we report default ORs from procedure output. ORs for factors involved in interactions with place of residence were calculated with the STATA Lincom postestimation command (6). The results from this model feed three tables presented in the paper that group risk factors for SARS-CoV-2 IgG antibodies (past infections) based on the socio-demographic characteristics of the

respondents (**Table 1**), household-related exposures (**Table 2**), and finally work-related and other risk factors for SARS-CoV-2 IgG antibodies (**Table 3**).

Supplementary **table S6**. Final multivariable logistic regression model used for estimating adjusted ORs

Factor:	Coef.	Std. Err.	t	P>t	[95% Conf. Interval]
Place of residence					
Areas of 50,000 pop. and over	(base)				
Areas under 50,000 pop	0.472	0.196	2.410	0.016	0.088; 0.856
Age group					
20-39	(base)				
40-59	0.291	0.092	3.160	0.002	0.110; 0.472
60-69	0.589	0.118	4.980	0.000	0.357; 0.820
70+	1.037	0.160	6.480	0.000	0.723; 1.351
Sex					
Male	(base)				
Female	-0.047	0.095	-0.490	0.622	-0.232; 0.139
Region					
1.Lower Silesian	(base)				
2.Kuyavian-Pomeranian	0.089	0.224	0.400	0.693	-0.351; 0.528
3.Lublin	0.363	0.200	1.810	0.070	-0.030; 0.755
4.Lubusz	0.148	0.242	0.610	0.542	-0.327; 0.622
5.Łódź	0.215	0.192	1.120	0.263	-0.161; 0.591
6.Lesser Poland	0.054	0.173	0.310	0.756	-0.285; 0.393
7.Masovian	-0.040	0.158	-0.260	0.799	-0.351; 0.270
8.Opole	0.046	0.246	0.190	0.853	-0.437; 0.528
9.Subcarpathian	0.438	0.202	2.170	0.030	0.042; 0.834
10.Polaskie	0.366	0.216	1.700	0.090	-0.057; 0.789
11.Pomeranian	0.239	0.187	1.280	0.200	-0.127; 0.606
12.Silesian	0.123	0.171	0.710	0.475	-0.214; 0.459
13.Holy Cross	0.565	0.215	2.630	0.009	0.144; 0.986
14.Warmian–Masurian	0.554	0.211	2.630	0.009	0.141; 0.967
15.Greater Poland	-0.134	0.184	-0.730	0.466	-0.494; 0.226
16.West Pomeranian	-0.243	0.213	-1.140	0.254	-0.660; 0.174
Number of household members					
Continuous	0.217	0.042	5.150	0.000	0.134; 0.299
Work during restrictions (since March 2020)					
No	(base)				
Yes	0.336	0.096	3.480	0.001	0.147; 0.525
Mainly remote work (during restrictions)					
No	(base)				
Yes	-0.492	0.120	-4.110	0.000	-0.726; -0.257
Contact with a confirmed case (since March 2020)					
No	(base)				
Yes	0.785	0.077	10.210	0.000	0.634; 0.936
COVID-19 vaccination status					
At least one dose	(base)				
Not vaccinated	1.000	0.099	10.090	0.000	0.806; 1.195
Interaction terms:					
Sex # Place of residence	0.341	0.140	2.440	0.015	0.067; 0.616
Number of household members # Place of residence	-0.180	0.057	-3.150	0.002	-0.292; -0.068
_cons	-3.153	0.237	-13.320	0.000	-3.617; -2.689

Impact of weights on estimated seroprevalence

Application of weights lowered the overall seroprevalence estimates marginally, from 31.6% to 29.8% (95% CI: 28.4–31.2), but had no impact on the observed rural-urban gradient and other trends seen whether weighted or unweighted data. Supplementary **table S7** shows the replication of the main results calculated on unweighted (raw) data. The t-tests were used to compare coefficients across these regressions.

Supplementary **table S7**. Replication of main results from Tables 1-3 without Weights, w_k

Subset:	Factor:	Seroprevalence estimates	Weighted AOR (95%CI)	Unweighted AOR (95%CI)	Coef. Difference	Std. Err.	t	P>|t|	[95% Conf. Interval]
Total	Age group (years)								
	20-39	29.1%	ref.	ref.					
	40-59	32.9%	1.34 (1.12-1.60)	1.39 (1.19-1.62)	-0.037	0.019	-1.920	0.055	-0.075; 0.001
	60-69	26.4%	1.80 (1.43-2.27)	2.04 (1.67-2.50)	-0.126	0.038	-3.330	0.001	-0.200; -0.052
	70+	28.0%	2.82 (2.06-3.86)	2.93 (2.26-3.78)	-0.036	0.023	-1.570	0.116	-0.081; 0.009
	Work during restrictions								
	Yes	31.6%	1.40 (1.16-1.69)	1.27 (1.09-1.49)	0.093	0.007	13.320	<0.001	0.079; 0.107
	No	27.0%	ref.	ref.					
	Mainly remote work								
	Yes	21.1%	0.61 (0.48-0.77)	0.68 (0.56-0.83)	-0.110	0.025	-4.410	<0.001	-0.159; -0.061
	No	30.8%	ref.	ref.					
	Contact with a known COVID case								
	Yes	42.4%	2.19 (1.89-2.55)	2.18 (1.93-2.47)	0.006	0.009	0.630	0.531	-0.013; 0.024
	No	24.8%	ref.	ref.					
	Received at least one dose of vaccine								
	No	34.7%	2.72 (2.24-3.30)	2.85 (2.45-3.32)	-0.048	0.019	-2.470	0.014	-0.085; -0.010
	Yes	18.6%	ref.	ref.					
	Living in Eastern Poland (univariate OR)								
	Yes	38.0%	1.61 (1.37-1.89)	1.45 (1.28-1.64)	0.106	0.005	19.680	<0.001	0.095; 0.116
	No	27.6%	ref.	ref.					
Areas under 50,000 pop.									
	Sex								
	Females	34.4%	1.34 (1.10-1.65)	1.29 (1.09-1.53)	0.041	0.017	2.460	0.014	0.008; 0.074
	Males	29.7%	ref.	ref.					
	One additional resident in a household		1.04 (0.95-1.13)	1.05 (0.98-1.13)	-0.015	0.008	-1.830	0.068	-0.031; 0.001
Areas of 50,000 pop. and over									
	Sex								
	Females	25.4%	0.95 (0.79-1.15)	0.94 (0.8-1.11)	0.013	0.025	0.510	0.611	-0.037; 0.063
	Males	27.9%	ref.	ref.					
	One additional resident in a household		1.24 (1.14-1.35)	1.23 (1.15-1.33)	0.007	0.008	0.950	0.341	-0.008; 0.023
Females									
	Place of residence								
	Areas under 50,000 pop.	34.4%	2.26 (1.58-3.21)	2.01 (1.49-2.71)	0.117	0.038	3.050	0.002	0.042; 0.191
	Areas of 50,000 pop. and over	25.4%	ref.	ref.					
Males									
	Place of residence								
	Areas under 50,000 pop	29.7%	1.60 (1.09-2.35)	1.47 (1.06-2.04)	0.088	0.036	2.450	0.014	0.018; 0.159
	Areas of 50,000 pop. and over	27.9%	ref.	ref.					

Impact of exclusion of laboratory borderline results on rural-urban gradient

We considered the worst-case scenario, in which all borderline records have been retained in the analysis and recoded against the expected gradient, i.e., borderline results in rural areas or small towns are treated as negatives, whereas they are treated as positives in larger cities. Our aim was to determine how the exclusion of borderline laboratory results could have affected the reported findings of the rural-urban gradient.

There were 228 records with borderline laboratory results, 106 of which were residents of rural regions or small towns with less than 50,000 residents and 122 of which were residents of mid-sized or large cities with 50,000 or more inhabitants.

The observed rural-urban gradient and other trends were consistent regardless of the subsets of the data. Supplementary **table S8** shows the replication of the main results calculated in this scenario. The t-tests were used to compare coefficients across two regressions.

Supplementary **table S8**. Replication of main results from Tables 1-3 with borderline laboratory results

Subset:	Factor:	Seroprev.. estimates	AOR (95%CI) excluded	AOR (95%CI) not excluded*	Coef. Difference	Std. Err.	t	P>|t|	[95% Conf. Interval]
Total	Age group (years)								
	20-39	29.1%	ref.	ref.					
	40-59	32.9%	1.34 (1.12-1.60)	1.32 (1.11-1.57)	0.016	0.008	1.940	0.052	0.000; 0.032
	60-69	26.4%	1.80 (1.43-2.27)	1.69 (1.35-2.11)	0.066	0.009	7.120	<0.001	0.048; 0.084
	70+	28.0%	2.82 (2.06-3.86)	2.59 (1.90-3.52)	0.087	0.012	7.090	<0.001	0.063; 0.111
	Work during restrictions								
	Yes	31.6%	1.40 (1.16-1.69)	1.34 (1.11-1.61)	0.043	0.007	6.330	<0.001	0.030; 0.056
	No	27.0%	ref.	ref.					
	Mainly remote work								
	Yes	21.1%	0.61 (0.48-0.77)	0.62 (0.49-0.77)	-0.007	0.017	-0.410	0.683	-0.040; 0.026
	No	30.8%	ref.	ref.					
	Contact with a known COVID case								
	Yes	42.4%	2.19 (1.89-2.55)	2.11 (1.82-2.45)	0.036	0.006	6.100	<0.001	0.025; 0.048
	No	24.8%	ref.	ref.					
	Received at least one dose of vaccine								
	No	34.7%	2.72 (2.24-3.30)	2.83 (2.34-3.43)	-0.041	0.009	-4.390	<0.001	-0.059; -0.023
	Yes	18.6%	ref.	ref.					
	Living in Eastern Poland (univariate OR)								
	Yes	38.0%	1.61 (1.37-1.89)	1.55 (1.32-1.81)	0.038	0.003	11.250	<0.001	0.032; 0.045
	No	27.6%	ref.	ref.					
Areas under 50,000 pop.									
	Sex								
	Females	34.4%	1.34 (1.10-1.65)	1.30 (1.06-1.59)	0.032	0.006	5.740	<0.001	0.021; 0.042
	Males	29.7%	ref.	ref.					
	One additional resident in a household		1.04 (0.95-1.13)	1.01 (0.93-1.10)	0.025	0.003	9.860	<0.001	0.020; 0.030
Areas of 50,000 pop. and over									
	Sex								
	Females	25.4%	0.95 (0.79-1.15)	0.95 (0.79-1.13)	0.010	0.011	0.880	0.381	-0.012; 0.031
	Males	27.9%	ref.	ref.					
	One additional resident in a household		1.24 (1.14-1.35)	1.23 (1.14-1.33)	0.008	0.005	1.540	0.123	-0.002; 0.019
Females									
	Place of residence								
	Areas under 50,000 pop.	34.4%	2.26 (1.58-3.21)	2.01 (1.42-2.84)	0.117	0.018	6.510	<0.001	0.082; 0.152
	Areas of 50,000 pop. and over	25.4%	ref.	ref.					
Males									
	Place of residence								
	Areas under 50,000 pop	29.7%	1.60 (1.09-2.35)	1.46 (1.00-2.12)	0.095	0.018	5.260	<0.001	0.059; 0.130
	Areas of 50,000 pop. and over	27.9%	ref.	ref.					

* all borderline laboratory results have been retained

References

1. Horvitz DG, Thompson DJ. A generalization of sampling without replacement from a finite universe. Journal of the American Statistical Association 1952; 47:663– 685.
2. Little RJ, Vartivarian S. On weighting the rates in non-response weights. Stat Med. 2003 May 15;22(9):1589-99. doi: 10.1002/sim.1513.
3. eHealth Centre. [Central e-registration for COVID-19 vaccine]. In Polish. Available at https://www.cez.gov.pl/index.php/pl/nasze-produkty/e-zdrowie-p1/nasze-produkty/e-zdrowie-p1/centralna-e-rejestracja-na-szczepienie-przeciw-covid-19, accessed 27.02.2023
4. Dugoff EH, Schuler M, Stuart EA. Generalizing observational study results: applying propensity score methods to complex surveys. Health Serv Res. 2014 Feb;49(1):284-303. doi: 10.1111/1475-6773.12090.
5. Lee BK, Lessler J, Stuart EA. Weight trimming and propensity score weighting. PLoS One. 2011 Mar 31;6(3):e18174. doi: 10.1371/journal.pone.0018174.
6. Svy postestimation — Postestimation tools for svy. Available at: https://www.stata.com/manuals/svysvypostestimation.pdf#svysvypostestimation
